# Neurological and Neurodegenerative Disorders: Novel Concepts and Treatment

**DOI:** 10.14336/AD.2021.0530

**Published:** 2021-07-01

**Authors:** Batool F Kirmani, Lee A Shapiro, Ashok K Shetty

**Affiliations:** ^1^Comprehensive Epilepsy and Functional Neurosurgery Program, Endovascular Therapy & Interventional Stroke Program, Department of Neurology, St. Joseph Bryan Regional Hospital, CHI St. Joseph Health, Bryan, TX, USA.; ^2^Texas A&M University College of Medicine, College Station, TX, USA.; ^3^Department of Neuroscience and Experimental Therapeutics, Texas A&M University College of Medicine, Brayan, TX, USA.; ^4^Institute for Regenerative Medicine, Department of Molecular and Cellular Medicine, Texas A&M University College of Medicine, College Station, TX, USA.

**Keywords:** neurological, neurodegenerative disorders, concepts, treatment

## Abstract

The journal “Aging and Disease” has released a special issue focused on novel concepts in understanding the pathophysiology and treatment of neurological and neurodegenerative disorders. The special issue comprises review and original research articles discussing the various disease mechanisms and/or treatment updates on aging, mild cognitive impairment, dementia, acute stroke, pediatric stroke, super-refractory status epilepticus, reflex epilepsy, drug-resistant epilepsy, Parkinson’s disease, and traumatic brain injury. This editorial discusses the highlights from these articles.

The expenditure for patients with the most common neurological and neurodegenerative disorders is a huge burden to society worldwide, exceeding $800 billion annually in the USA alone [1]. The overall cost is estimated to surge exponentially in the coming years as the elderly population would double by 2050. The expenses of dementia and stroke alone are estimated to total over $600 billion by 2030 [1]. Therefore, comprehending the causes, pathophysiology, prognosis, and efficacy of novel treatment strategies for these diseases have broad implications for reducing the burden and improving clinical management [2]. In this special issue, experts in their respective fields have provided authoritative reviews, updates, perspectives, or original research findings on mechanisms underlying neurological and neurodegenerative diseases, or novel treatment approaches to combat aging, mild cognitive impairment, dementia, acute stroke, pediatric stroke, super-refractory status epilepticus, reflex epilepsy, drug-resistant epilepsy, Parkinson’s disease, and traumatic brain injury.

Mild cognitive impairment (MCI) is a condition between the expected cognitive decline associated with physiological aging and the more severe dementia [3]. MCI is typified by noticeable impairments in memory recall, language, or judgment. Such changes might result in missing appointments, losing the train of thought, impulsive behavior, frequent irritability, depression, anxiety, and apathy. While symptoms of MCI remain stable or improve over the years in most, a significant percentage of patients will progress into Alzheimer’s disease or another kind of dementia. Dr. Mattos and colleagues described a relationship in older patients with mild cognitive impairments and insomnia. The authors discussed the benefits and disadvantages of the two treatments used to treat insomnia, including cognitive behavioral therapy and pharmacotherapy [4]. The review highlighted that more widely used pharmacotherapy has short-term effectiveness but is associated with recurrence, ineffectiveness after chronic use, and adverse side effects. A perspective on metabolic insufficiency underlying aging and dementia by Turner discussed several metabolic treatment approaches but pointed out that surrogate biomarkers of metabolism are required to make dynamic estimates of neuronal demand, the sufficiency of neurovascular coupling, and glymphatic flow in patients afflicted with aging, MCI, or dementia [5]. Such biomarkers might also be used to gauge the efficacy on various metabolic treatments to slow down aging or the progression of dementia [5]. Dementia, a condition exemplified by memory loss, problems with language, problem-solving or and thinking abilities, adversely impacts daily life. Nizamutdinov and associates discuss data on the safety and potential therapeutic benefits of transcranial near-infrared (tNIR) light stimulations in the treatment of dementia. They reported that low power tNIR light stimulations with an active photobiomodulation for 6 minutes twice daily for eight weeks could improve cognitive function, with many patients reporting improved sleep as well [6].

Stroke, one of the prominent causes of morbidity and mortality, leads to long-term disability in most patients [7]. Hollist and co-authors discussed the recent updates of acute ischemic stroke management regarding mechanical thrombectomy as well as thrombolytics, including a single-bolus thrombolytic or clot-busting agent Tenecteplase [8]. Clinical trials were discussed concerning the therapeutic window and last known normal prior to the stroke symptom onset. Risks and benefits of both acute and surgical management were reviewed in terms of improvement in disability and risk of intracerebral hemorrhage. The article highlighted that future studies need to determine the efficacy with direct comparisons between different innovative treatments and continue to advance the boundaries of acute ischemic stroke care. In another article, Hollist and associates reviewed recent updates on the treatment of pediatric stroke [9]. Pediatric Stroke is a rare type of stroke in which research is still underway. The authors discussed the epidemiology of pediatric stroke, etiologies, current clinical trials, clinical presentation, acute treatment with IV thrombolytics and mechanical thrombectomy, and preventative management.

Super-refractory status epilepticus (SRSE) is a life-threatening neurological emergency with high morbidity and mortality [10]. Kirmani and colleagues discussed the management of SRSE, including traditional and alternative anesthetic agents with antiepileptic agents. The alternative therapies, including hypothermia, steroids, immunosuppressive agents, electrical and magnetic stimulation therapies, emergent resective epilepsy surgery, the ketogenic diet, pyridoxine infusion, cerebrospinal fluid drainage, and magnesium infusion, were also conferred for this devastating illness [11]. Epilepsy afflicts ~70 million people worldwide and contributes substantially to the burden of neurological disorders. While antiepileptic drugs (AEDs) effectively control seizures in most epilepsy patients, ~30-40% of patients develop pharmaco-resistant epilepsy [12-14]. Drug-resistant epilepsy patients who are not resective surgery candidates can benefit from alternative treatment modalities, including neuromodulation. Rincon and co-authors provided an in-depth review of the current evidence, indications, safety considerations, and side effects of the neuromodulation therapies such as vagal nerve stimulation (VNS), responsive neurostimulation (RNS), or deep brain stimulation (DBS) for drug-resistant epilepsy [15]. Hanif and Musick discussed the pathophysiology and treatment of reflex epilepsy, characterized by epileptic events elicited in response to a specific afferent stimulus, which may comprise various intrinsic and extrinsic factors, including flashing lights or reading a book. Reflex seizures start by stimulating functional anatomic networks associated with physiological activities and overlapping with regions of cortical hyperexcitability. The authors highlighted that treatment should include antiseizure medication and lifestyle modifications. They also suggested surgical interventions when amenable [16]. In another article, Kipnis and Kadam discussed novel concepts for the role of chloride transporters in refractory seizures emerging after hypoxic-ischemic encephalopathy (HIE) or arterial ischemic stroke in the pediatric population. Such seizures are typically associated with intellectual disability and neurocognitive comorbidities. The authors pointed out that type 2 K^+^-Cl^-^ cotransporter (KCC2), the chloride extruder in neurons enabling potent hyperpolarizing synaptic inhibition in the brain, is one of the main culprits in the pathophysiology underlying HIE and arterial ischemic stroke-induced refractory seizures. The authors discussed preclinical research that suggested that manipulating KCC2 could potentially eliminate seizures in HIE and temporal lobe epilepsy [17].

Parkinson’s disease (PD), the second most frequent neurodegenerative disease in the aging population, is characterized by the degeneration of neurons in the substantia nigra pars compacta region and other areas of the brain [18-19]. PD currently affects ~7 million people worldwide that is expected to increase to 14 million by 2040. A review article in the special issue has discussed the current diagnosis and treatment approaches for motor and non-motor symptoms as well as significant advancements and updates in the medical and surgical management of PD [20]. An original research article in this special issue demonstrates how the loss of nuclear factor (erythroid-derived 2)-like 2 (NRF2) worsens Parkinsonian pathology and behavioral deficits in human α-synuclein overexpressing mice, which is highly relevant to understanding PD pathophysiology [21]. NRF-2, a master regulator of oxidative stress, promotes cellular defense and survival mechanisms after activation. Its loss is associated with increased oxidative and proteotoxic stress. Using a series of studies, the authors have demonstrated that NRF-2 loss leads to increased phosphorylation and oligomerization of α-Syn, associated with dopaminergic neuron loss, greater oxidative stress and neuroinflammation, and more pronounced behavioral deficits. Overall, the findings point to NRF-2 as a target to slow down PD pathogenesis [21]. A review by Huntington and Srinivasan discussed the suitability of an animal model employing adeno-associated virus-mediated expression of α-syn as a tool to understand PD pathogenesis. The authors discussed AAV vectors relevant to modeling a-syn dysfunction in rodent prototypes of PD, the role of astrocytes in propagating a-syn pathology, and the promise of AAVs as a tool to comprehend the role of astrocytes in α-syn pathology during PD progression [22].

Another study in the special issue demonstrated the various adverse effects of 7-day pyruvate administration after TBI [23]. Using a lateral fluid percussion model of TBI, the authors reported that pyruvate treatment after TBI worsened behavioral and motor responses, increased gliosis and neurodegeneration and decreased myelin repair. The authors cautioned that pyruvate is not an ideal drug for improving brain function in the acute phase of TBI.
